# The complete plastome of *Amaranthus roxburghianus* (Amaranthaceae)

**DOI:** 10.1080/23802359.2024.2378996

**Published:** 2024-07-16

**Authors:** Liqiang Wang, Xiaohan Zhang, Hongqin Li, Shu Wang

**Affiliations:** College of Pharmacy, Heze University, Heze, Shandong Province, P. R. China

**Keywords:** *Amaranthus roxburghianus*, Amaranthaceae, plastome, phylogenetic analysis, Next-generation sequencing technology

## Abstract

*Amaranthus roxburghianus* H.W. Kung 1935, belonging to the Amaranthaceae family, is recognized for its significant medicinal properties. However, molecular research on this species has been limited. This study represents the inaugural documentation of the sequencing and assembly of the complete plastome of *A. roxburghianus*. The genome spans a total length of 149,969 base pairs (bp), exhibiting a conventional quadripartite structure. This structure comprises a large single-copy (LSC) region of 83,917 bp, a small single-copy (SSC) region of 18,124 bp, and two inverted repeat (IR) regions, each extending to 23,964 bp. In its entirety, the *A. roxburghianus* plastome encompasses 128 genes, of which 107 are unique, encompassing 77 individual protein-coding genes, 26 unique tRNA genes, and four unique rRNA genes. Phylogenetic analysis has shown a close resemblance between *A. roxburghianus* and *A. polygonoides*, both part of the subgenus *Albersia*. Although the genus *Amaranthus* is roughly divided into three subgenera, additional plastid genomic data are required for a more accurate assignment of *A. albus* and *A. blitoides*. The sequencing of this plastome is a significant step forward, likely to expedite the development of molecular markers and significantly contribute to genetic assays involving this distinctive species.

## Introduction

*Amaranthus*, a genus within the family Amaranthaceae, includes approximately 70 species of herbaceous plants with a global distribution. These plants are generally annual or perennial, featuring monoecious flowers and leaves of diverse shapes. The *Amaranthus* genus holds significant economic and cultural value, being utilized in food, medicine, and decoration. Additionally, these plants have been extensively researched for their potential in environmental remediation and biofuel production, among other applications (Kongdang et al. 2021). In classical taxonomy, *Amaranthus* is roughly categorized into three subgenera: subgenus *Albersia*, subgenus *Amaranthus*, and subgenus *Acnida* (Xu et al. 2020a). The plastome plays a crucial role in the phylogenetic study of the *Amaranthus* genus. To date, the nuclear genome of five *Amaranthus* species has been resolved, and the plastomes of 16 *Amaranthus* species have been sequenced.

*Amaranthus roxburghianus* H.W. Kung 1935 is an annual herb in the Amaranthaceae family. Traditionally, its roots have been used to treat colic pains. Recent research indicates that the root extract of *A. roxburghianus*, particularly when combined with piperine, may be effective in treating ulcerative colitis in mice. While further studies are necessary to comprehensively understand its medicinal properties, *A. roxburghianus* demonstrates potential as a natural remedy for certain health conditions (Nirmal et al. 2013).

Recent research has primarily focused on the medicinal value of *Amaranthus roxburghianus*, especially its pharmacological activities and the isolation of compounds (Nirmal et al. 2013). However, there has been a scarcity of molecular research on this species. Understanding its genetic diversity is crucial for the conservation of *Amaranthus* and for elucidating its evolutionary history. To contribute additional genetic data and ascertain the phylogenetic position of *A. roxburghianus* within the *Amaranthus* genus, we have sequenced and characterized its complete plastome. This effort aims to support further evolutionary research within the genus.

## Materials

**Figure 1.**
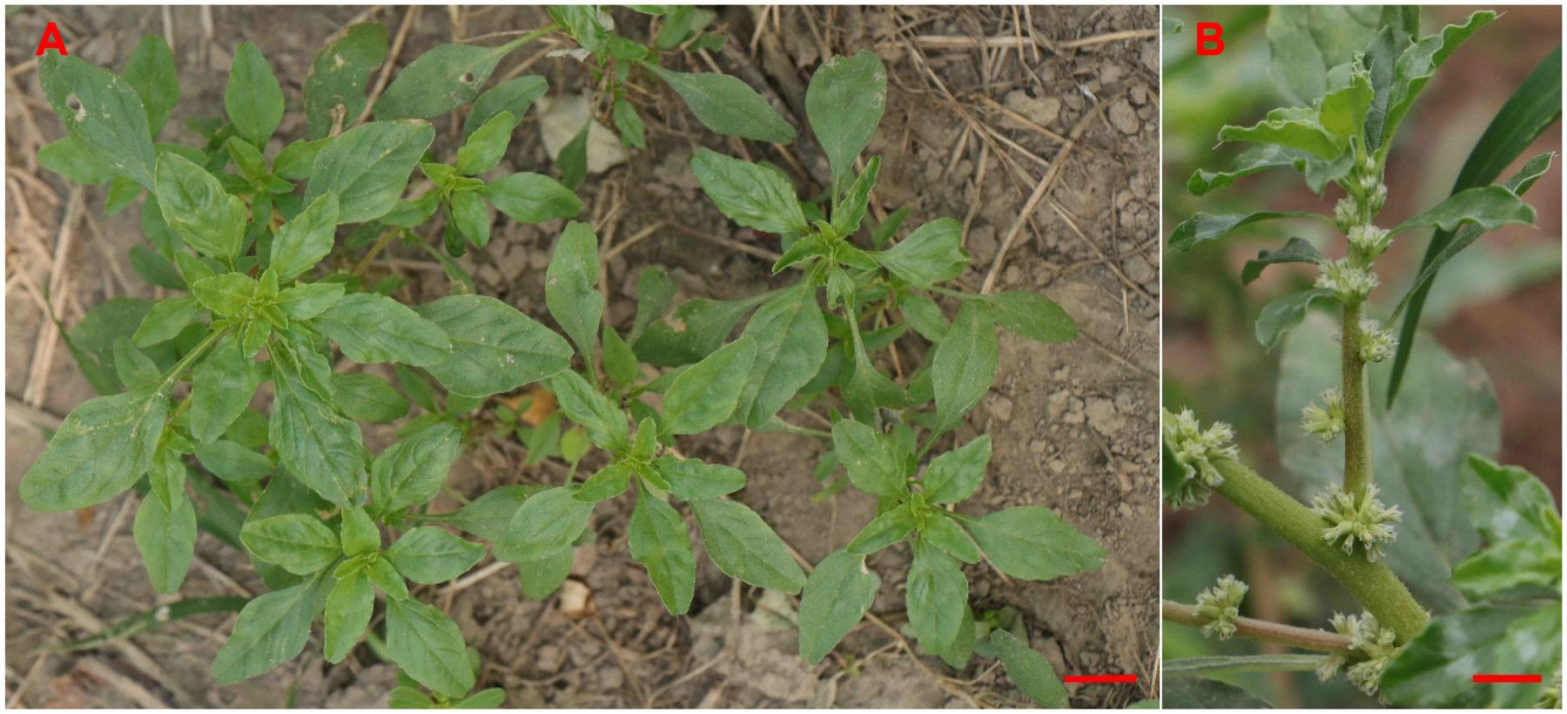
Panorama (A) and detail (B) photos of *Amaranthus roxburghianus*. Photo by Liqiang Wang, plant coordinates 35°16′23.65'' N, 115°27′32.85'' E. Main identifying features: stem erect, light green, 30-65 cm, much branched, glabrous. Petiole 1-2.5 cm, slender; leaf blade ovate-rhombic, obovate, or oblong, 2-5 × 1-2.5 cm, base cuneate, margin undulate, apex notched, mucronate. Flowers few, sparsely clustered in axils. Bracts and bracteoles subulate, ca. 2 mm, abaxially with distinct midvein, apex long pointed. Tepals lanceolate, ca. 2.5 mm, apex acuminate, long pointed. Stamens shorter than perianth; stigmas 3. Utricles ovoid, subequal to perianth, ca. 3 mm, circumscissile. Seeds brownish black, subglobose, ca. 1 mm in diameter. The scale of the scale bar is 10 cm.

The fresh leaves of *A. roxburghianus*, depicted in Figure 1, utilized in this study were collected from Heze University, located in Heze City, Shandong Province, China (115° 27′ 32.85" E, 35° 16′ 23.65" N). Voucher samples of this species are preserved in the specimen room of Heze University (voucher number: HZ220808; contact: Hongqin Li; email: 463056627@qq.com). The species was identified by Hongqin Li.

## Methods

We extracted total DNA from the fresh leaves of *A. roxburghianus* using a Plant Genomic DNA Kit (Tiangen, China) and sequenced it on the HiSeq2500 platform at Wuhan Benagen Technology Company Limited, Wuhan, China. We filtered the raw reads by eliminating adapters and low-quality bases by Trimmomatic (v0.35) (Bolger et al. 2014). After this filtering process, approximately 13.8 GB of clean reads were assembled using GetOrganelle (v1.7.1) (Jin et al. 2020). After assembly, we verified the accuracy of the assembled plastome using minimap2 (Li 2018) in conjunction with samtools (Li et al. 2009). Subsequently, we annotated the plastome by CPGAVAS2 (Shi et al. 2019).

To establish the phylogenetic relationship of *A. roxburghianus*, we utilized PhyloSuite (Zhang et al. 2020) to download the plastomes of 32 other *Amaranthus* species from GenBank. Additionally, plastomes of *Celosia cristata* (MK470118) and *C. argentea* (MZ636551) were downloaded to serve as outgroups. We aligned the entire plastome sequences using MAFFT software (https://mafft.cbrc.jp/alignment/software/) with default parameters (Katoh and Standley 2013). Subsequently, a maximum-likelihood (ML) phylogenetic tree was constructed using IQ-TREE (v2.0) (Nguyen et al. 2015), employing the Best-fit model of TVM + F + I + I+R4 and incorporating 1000 bootstrap replicates.

## Results

**Figure 2.**
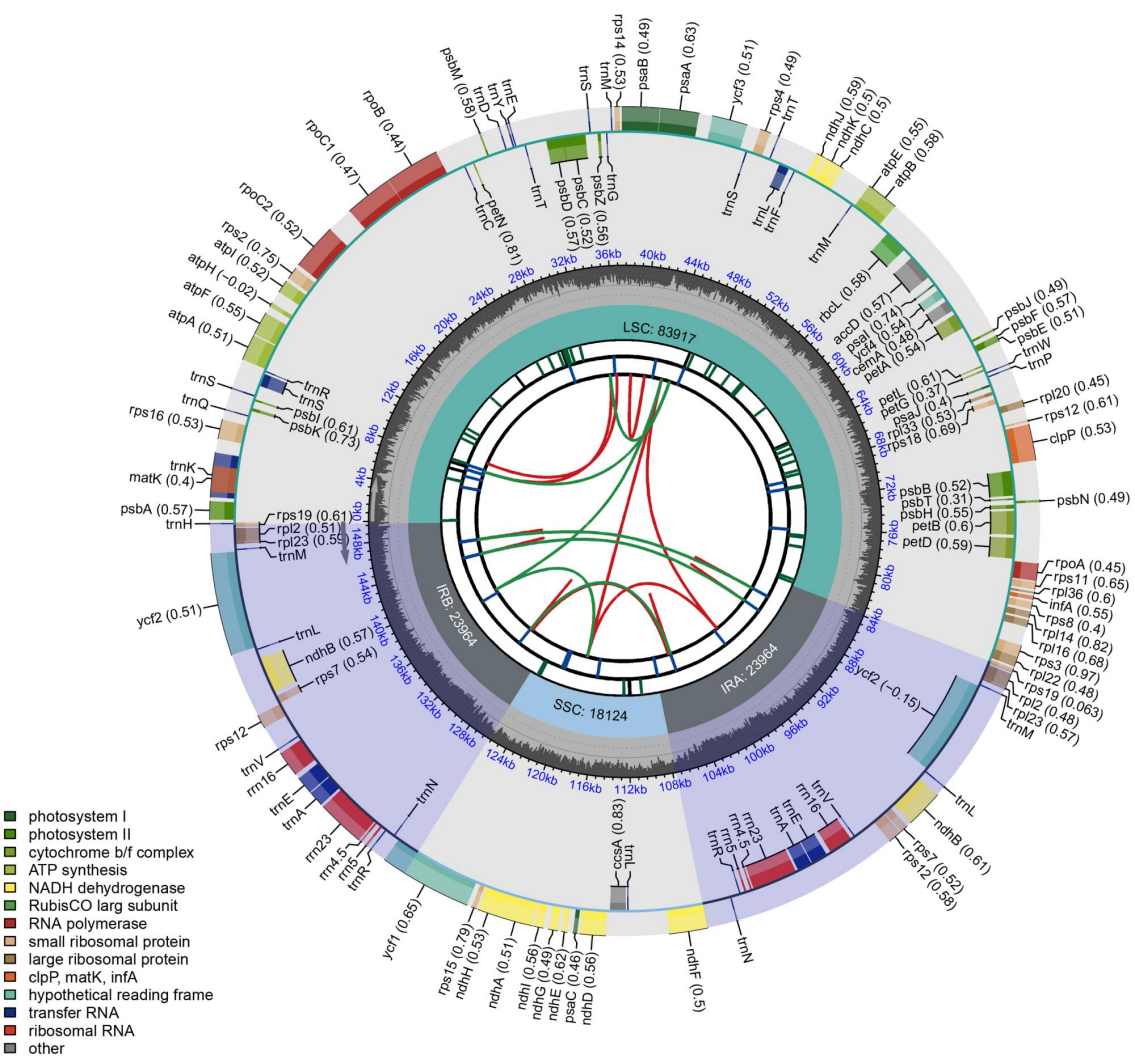
The schematic map of the *Amaranthus roxburghianus* plastome. The species name is shown at the top left of the map. The map displays six tracks representing various genetic elements. Track one illustrates dispersed repeats, with direct and palindromic repeats connected by red and green arcs. Long tandem repeats in track two are shown as short blue bars. Track three depicts short tandem repeats/microsatellite sequences, differentiated by colored bars: black for complex repeats and green, yellow, purple, blue, orange, and red for repeat units of sizes 1 to 6, respectively. The fourth track indicates single-copy and inverted repeat regions, while the fifth track presents the genome’s GC content. The sixth track details genes, color-coded by functional classification and potentially including codon usage bias, with gene transcription direction indicated (inner genes clockwise, outer genes anticlockwise). The functional classification of genes is provided in the bottom left corner.

The plastome of *A. roxburghianus*, as deciphered in this study, is a circular DNA molecule with a total length of 149,969 base pairs (bp). The reliability of the genome assembly was strongly supported by the results of the mapping experiment, with a mean sequencing depth of 1821.4× (Figure S1). The genome exhibits a conventional quadripartite structure, comprising a large single-copy (LSC) region of 83,917 bp, a small single-copy (SSC) region of 18,124 bp, and a pair of inverted repeats (IR) regions, each of 23,964 bp. The overall GC content is 36.52%, which is lower than that of the IR regions (42.68%) but higher than that of the LSC (34.37%) and SSC regions (30.14%). The plastome contains 128 genes, of which 107 are unique, including 77 distinct proteins, 26 distinct tRNAs, and 4 distinct rRNA genes (Figure 2). Seven unique protein-coding genes (*atp*F, *ndh*A, *ndh*B (×2), *pet*B, *pet*D, *rpo*C1, and *rps*16) contain one intron, while three unique genes (*clp*P, *rps*12 (×2), and *ycf*3) contain two introns. The cis-splicing genes are *rps*16, *atp*F, *rpo*C1, *ycf*3, *clp*P, *pet*B, *pet*D, *ndh*B (×2), and *ndh*A, and the trans-splicing gene is *rps*12 (×2). The structures of the cis- and trans-splicing genes are illustrated in Figure S2. The combined length of the rRNA and tRNA genes constitutes 6.03% and 1.83% of the entire plastome, respectively. Seven tRNA genes (*trn*K-UUU, *trn*S-CGA, *trn*L-UAA, *trn*E-UUC (×2), and *trn*A-UGC (×2)) contain one intron.

**Figure 3.**
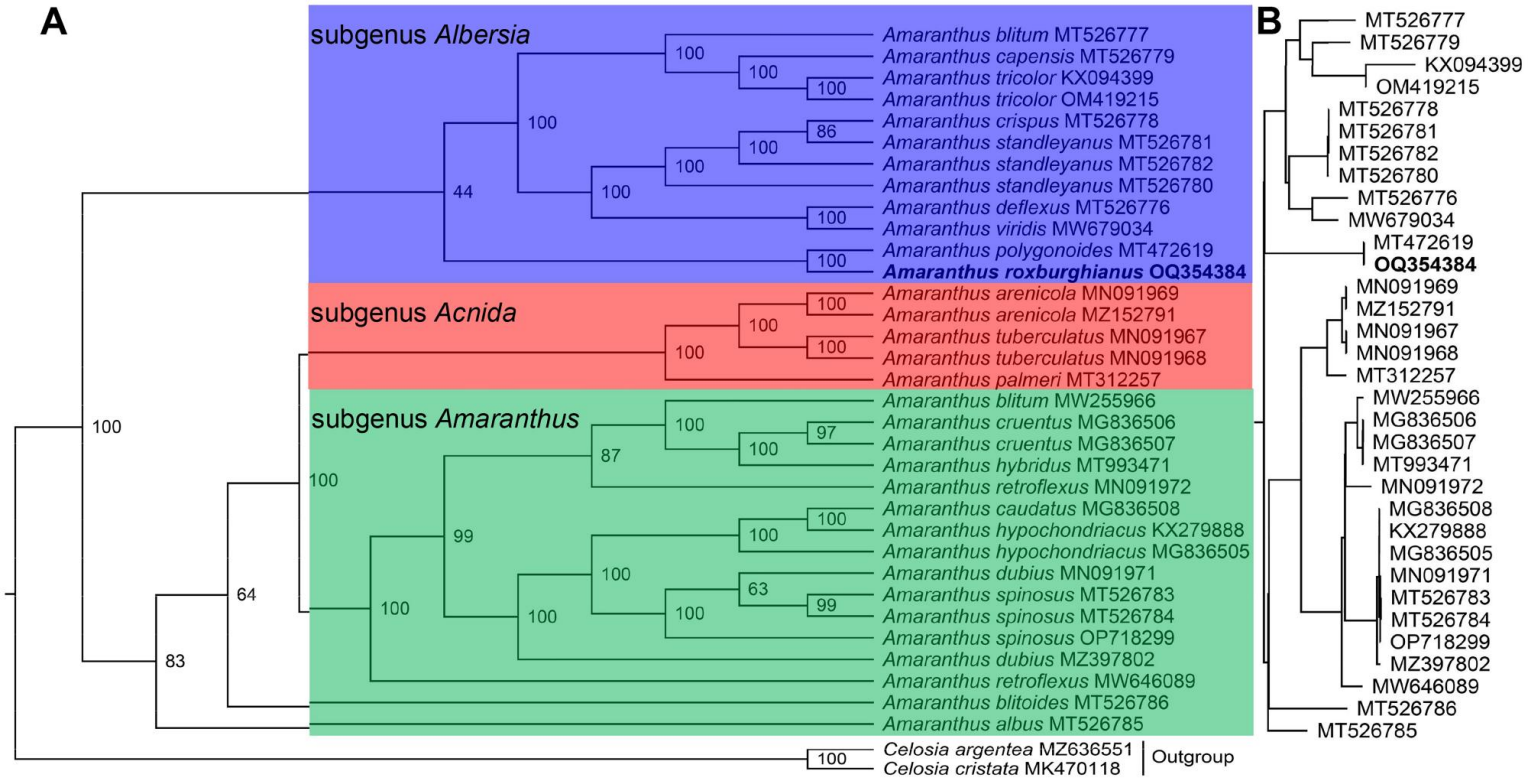
The maximum likelihood phylogeny of *Amaranthus roxburghianus* and its close relatives using whole plastome sequences. The bootstrap values based on 1000 replicates were shown on each node in the cladogram tree (a). The corresponding phylogram tree is shown in panel B without outgroups. The tree was constructed with 35 species, they were *A. albus* (MT526785) (Xu et al. 2022), *A. arenicola* (MN091969) (Xu et al. 2022), *A. arenicola* (MZ152791) (Xu et al. 2022), *A. blitoides* (MT526786) (Xu et al. 2022), *A. blitum* (MT526777) (Xu et al. 2021), *A. blitum* (MW255966), *A. capensis* (MT526779) (Xu et al. 2021), *A. caudatus* (MG836508), *A. cruentus* (MG836506), *A. cruentus* (MG836507), *A. deflexus* (MT526776) (Xu et al. 2021), *A. dubius* (MN091971) (Xu et al. 2022), *A. dubius* (MZ397802) (Xu et al. 2021), *A. hybridus* (MT993471) (Bai et al. 2021), *A. hypochondriacus* (KX279888), *A. hypochondriacus* (MG836505) (Xu et al. 2022), *A. palmeri* (MT312257) (Xu et al. 2022), *A. polygonoides* (MT472619) (Xu et al. 2022), *A. retroflexus* (MN091972) (Xu et al. 2022), *A. retroflexus* (MW646089) (Lou and Fan 2021), *A. roxburghianus* (OQ354384, this study), *A. spinosus* (MT526783) (Xu et al. 2022), *A. spinosus* (MT526784) (Xu et al. 2022), *A. spinosus* (OP718299) (Xu et al. 2022), *A. crispus* (MT526778) (Xu et al. 2021), *A. standleyanus* (MT526780) (Xu et al. 2021), *A. standleyanus* (MT526781) (Xu et al. 2021), *A. standleyanus* (MT526782) (Xu et al. 2021), *A. tricolor* (KX094399) (Xu et al. 2022), *A. tricolor* (OM419215) (Xu et al. 2022), *A. tuberculatus* (MN091967) (Xu et al. 2022), *A. tuberculatus* (MN091968) (Xu et al. 2022), *A. viridis* (MW679034) (Ding et al. 2021), *Celosia cristata* (MK470118, outgroup), and *C. argentea* (MZ636551, outgroup). Bootstrap supports were calculated from 1000 replicates. The *A. roxburghianus* was labeled in bold font in the phylogenetic tree. The blue, red and green box represents subgenus *Albersia*, subgenus *Acnida* and subgenus *Amaranthus* in the *Amaranthus.*

In the phylogenetic analysis, the maximum-likelihood (ML) phylogenetic tree comprises 33 nodes (excluding the root), among which 27 nodes have bootstrap values of not less than 97. This phylogenetic tree provides a dependable topological structure that indicates the evolutionary relationships among different *Amaranthus* species. According to the tree, *A. roxburghianus* and *A. polygonoides* form a monophyletic group with a bootstrap value of 100 (Figure 3), indicating their placement within the Subgenus *Albersia*.

## Conclusion and discussion

This study presents the primary characterization of the plastome of *A. roxburghianus* for the first time, which exhibits a typical annular tetrad structure with a size of 149,969 base pairs (bp) and contains 128 predicted genes. Compared with other reported plastomes of the *Amaranthus* genus (Lou and Fan 2021; Chaney et al. 2016; Xu et al. 2022; Xu et al. 2020b; Ding et al. 2021; Xu et al. 2021; Bai et al. 2021), the *A. roxburghianus* plastome shows a high degree of similarity in terms of plastome length and gene content.

The classification of the *Amaranthus* genus presents considerable challenges due to interspecific hybridization and gene introgression, leading to numerous intricate taxa that are challenging to delineate (Xu et al. 2022). Various authors have explored the taxonomy and evolution of this genus. In 2015, Bayón categorized monoecious species into three subgenera (Bayón 2015), while in 1955, Sauer (1955) classified dioecious species into three subgenera, drawing on both morphological and molecular biology data. Despite these efforts, some controversies still persist within the classification (Xu et al. 2022).

In this study, we reconstructed a phylogenetic tree based on an expanded set of plastome sequences. Our findings revealed that not only did the subgenus *Albersia* and the subgenus *Acnida* form monophyletic taxa, but the subgenus *Amaranthus* also exhibited a similar pattern. In Xu et al.'s report (2022), while the subgenus *Amaranthus* and subgenus *Acnida* were described as forming monophyletic taxa, the subgenus *Albersia* was not considered a monophyletic taxon. Contrary to Xu et al.'s reports (2022), our analysis suggests that *A. albus* and *A. blitoides*, which belong to the Galápagos Clade, do not fit within the subgenus *Albersia*. Instead, they should be tentatively classified under the subgenus Amaranthus. A significant finding of our study is that the genus *Amaranthus* can tentatively be divided into three subgenera. However, more plastid genomic data is required to determine the classification of *A. albus* and *A. blitoides*. Ultimately, these results lay a foundation for taxonomic revision, the understanding of phylogenetic evolution, the study of weed biology, and the development of genetic resources within the Amaranthus species.

## Supplementary Material

Supplemental Material

## Data Availability

The plastome sequence has been deposited in GenBank (https://www.ncbi.nlm.nih.gov/genbank/) with the accession number of OQ354384 (https://www.ncbi.nlm.nih.gov/nuccore/OQ354384). The associated BioProject, Bio-Sample and SRA numbers are PRJNA928567, SAMN36852648 and SRR23251385. (https://www.ncbi.nlm.nih.gov/sra/?term=SRR12620715).

## References

[CIT0001] Bai X, Ye X, Luo Y, Liu C, Wu Q. 2021. Characterization of the first complete chloroplast genome of *Amaranthus hybridus* (Caryophyllales: Amaranthaceae) with phylogenetic implications. Mitochondrial DNA B Resour. 6(11):3306–3308. doi:10.1080/23802359.2021.1994890. 34722881 PMC8555552

[CIT0002] Bayón ND. 2015. Revisión taxonómica de las especies monoicas de *Amaranthus* L. (Amaranthaceae): *Amaranthus* subg. *Albersia* y *Amaranthus* subg. *Albersia*. Ann Missouri Botanical Garden. 101(2):261–383. doi:10.3417/2010080.

[CIT0003] Bolger AM, Lohse M, Usadel B. 2014. Trimmomatic: a flexible trimmer for Illumina sequence data. Bioinformatics. 30(15):2114–2120. doi:10.1093/bioinformatics/btu170. 24695404 PMC4103590

[CIT0004] Chaney L, Mangelson R, Ramaraj T, Jellen EN, Maughan PJ. 2016. The complete chloroplast genome sequences for four *Amaranthus* species (Amaranthaceae). Appl Plant Sci. 4(9):1600063. apps. doi:10.3732/apps.1600063. PMC5033369 27672525

[CIT0005] Ding DB, Pang QH, Han XJ, Fan SJ. 2021. Characterization and phylogenetic analysis of the complete chloroplast genome of *Amaranthus viridis* (Amaranthaceae). Mitochondrial DNA B Resour. 6(9):2610–2612. doi:10.1080/23802359.2021.1961631. 34395893 PMC8354152

[CIT0006] Jin J-J, Yu WB, Yang JB, Song Y, dePamphilis CW, Yi TS, et al 2020. GetOrganelle: a fast and versatile toolkit for accurate *de novo* assembly of organelle genomes. Genome Biol. 21(1):241. doi:10.1186/s13059-020-02154-5. 32912315 PMC7488116

[CIT0007] Katoh K, Standley DM. 2013. MAFFT multiple sequence alignment software version 7: improvements in performance and usability. Mol Biol Evol. 30(4):772–780. doi:10.1093/molbev/mst010. 23329690 PMC3603318

[CIT0008] Kongdang P, Dukaew N, Pruksakorn D, Koonrungsesomboon N. 2021. Biochemistry of *Amaranthus* polyphenols and their potential benefits on gut ecosystem: a comprehensive review of the literature. J Ethnopharmacol. 281(281):114547. doi:10.1016/j.jep.2021.114547. 34425138

[CIT0009] Li H, Handsaker B, Wysoker A, Fennell T, Ruan J, Homer N, et al. 2009. The sequence alignment/map format and SAMtools. Bioinformatics. 25(16):2078–2079. doi:10.1093/bioinformatics/btp352. 19505943 PMC2723002

[CIT0010] Li H. 2018. Minimap2: pairwise alignment for nucleotide sequences. Bioinformatics. 34(18):3094–3100. doi:10.1093/bioinformatics/bty191. 29750242 PMC6137996

[CIT0011] Lou BX, Fan SJ. 2021. Characterization and phylogenetic analysis of the complete plastome of *Amaranthus retroflexus* L. (Amaranthaceae), an annual weeds. Mitochondrial DNA B Resour. 6(10):2847–2848. doi:10.1080/23802359.2021.1970640. 34514150 PMC8425639

[CIT0012] Nguyen LT, Schmidt HA, von Haeseler A, Minh BQ. 2015. IQ-TREE: a fast and effective stochastic algorithm for estimating maximum-likelihood phylogenies. Mol Biol Evol. 32(1):268–274. doi:10.1093/molbev/msu300. 25371430 PMC4271533

[CIT0013] Nirmal SA, Ingale JM, Pattan SR, Bhawar SB. 2013. *Amaranthus roxburghianus* root extract in combination with piperine as a potential treatment of ulcerative colitis in mice. J Integr Med. 11(3):206–212. doi:10.3736/jintegrmed2013022. 23570686

[CIT0014] Sauer J. 1955. Revision of the dioecious amaranths. Madroño. 13(1):5–46.

[CIT0015] Shi L, Chen H, Jiang M, Wang L, Wu X, Huang L, et al. 2019. CPGAVAS2, an integrated plastome sequence annotator and analyzer. Nucleic Acids Res. 47(W1):W65–W73. doi:10.1093/nar/gkz345. 31066451 PMC6602467

[CIT0016] Xu H, Pan X, Wang C, Chen Y, Chen K, Zhu S, et al. 2020a. Species identification, phylogenetic analysis and detection of herbicide‑resistant biotypes of *Amaranthus* based on ALS and ITS. Sci Rep. 10(1):11735.,. doi:10.1038/s41598-020-68541-x. 32678146 PMC7366686

[CIT0017] Xu H, Xiang N, Du W, Zhang J, Zhang Y. 2022. Genetic variation and structure of complete chloroplast genome in alien monoecious and dioecious *Amaranthus* weeds. Sci Rep. 12(1):8255. doi:10.1038/s41598-022-11983-2. 35585207 PMC9117656

[CIT0018] Xu XY, Wu JJ, Yan J, Zhou X, Wang RH, Qi ZC, et al 2020b. The complete chloroplast genome sequence of Palmer Amaranth (*Amaranthus palmeri* S. Watson). Mitochondrial DNA Part B. 5(2):1991–1992. doi:10.1080/23802359.2020.1756969.

[CIT0019] Xu XY, Yan J, Li HR, Feng YQ, Qi ZC, Yan XL. 2021. The complete chloroplast genome sequence of spleen amaranth (*Amaranthus dubius* Mart. ex Thell., Amaranthaceae). Mitochondrial DNA B Resour. 6(11):3267–3268. doi:10.1080/23802359.2021.1992318. 34712806 PMC8547846

[CIT0020] Zhang D, Gao F, Jakovlić I, Zou H, Zhang J, Li WX, et al 2020. PhyloSuite: an integrated and scalable desktop platform for streamlined molecular sequence data management and evolutionary phylogenetics studies. Mol Ecol Resour. 20(1):348–355. doi:10.1111/1755-0998.13096. 31599058

