# Supplementary material for: The complete plastome of Amaranthus roxburghianus (Amaranthaceae)
Source: Mitochondrial DNA B Resour. 2024 Jul 16;9(7):871–5. doi: 10.1080/23802359.2024.2378996 (PMC467102; doi:10.1080/23802359.2024.2378996)
Supplement: Supplemental Material [file TMDN_A_2378996_SM1105.docx]

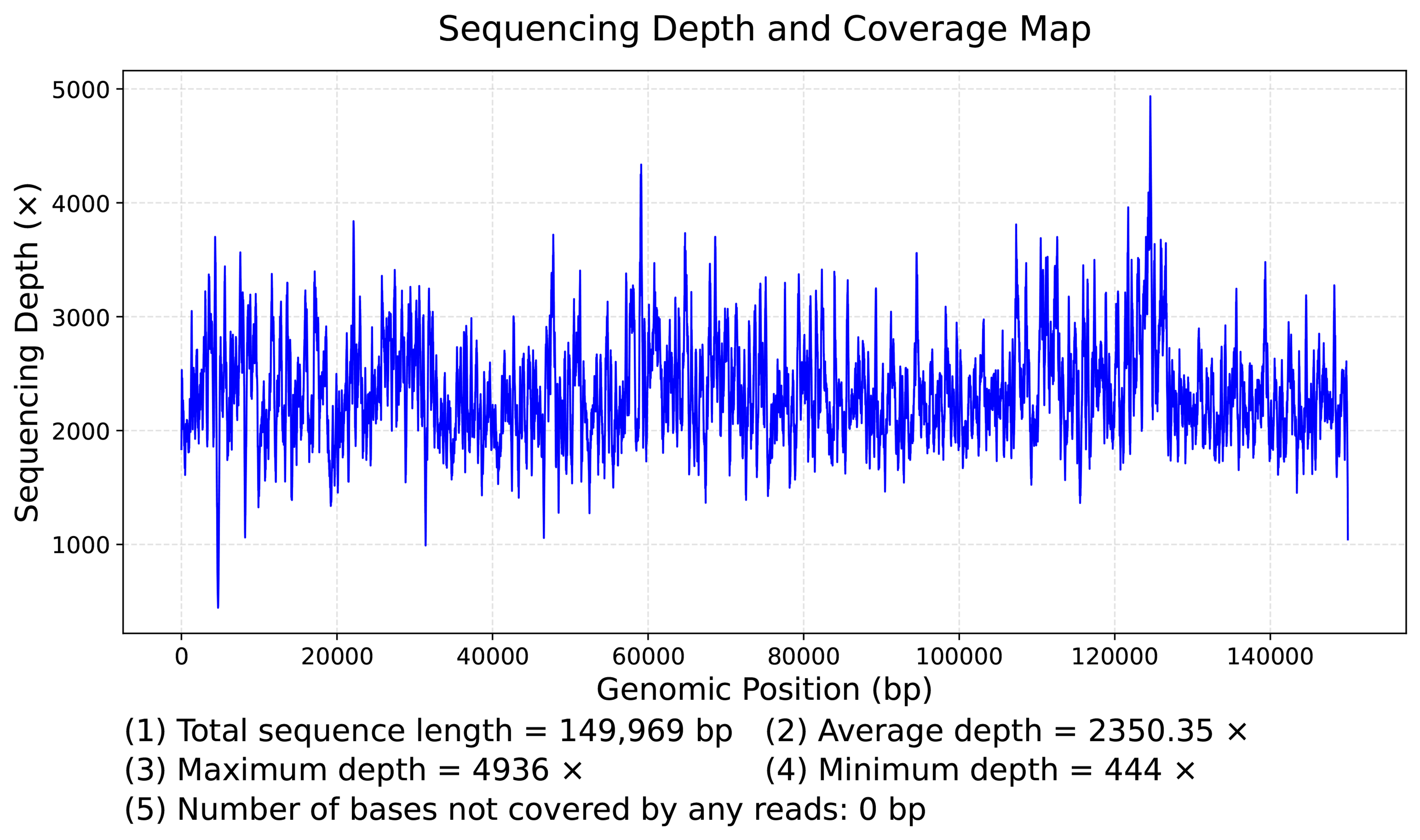


**Figure S1.** The sequencing depth and coverage map of the whole assembled *Amaranthus roxburghianus* plastome.


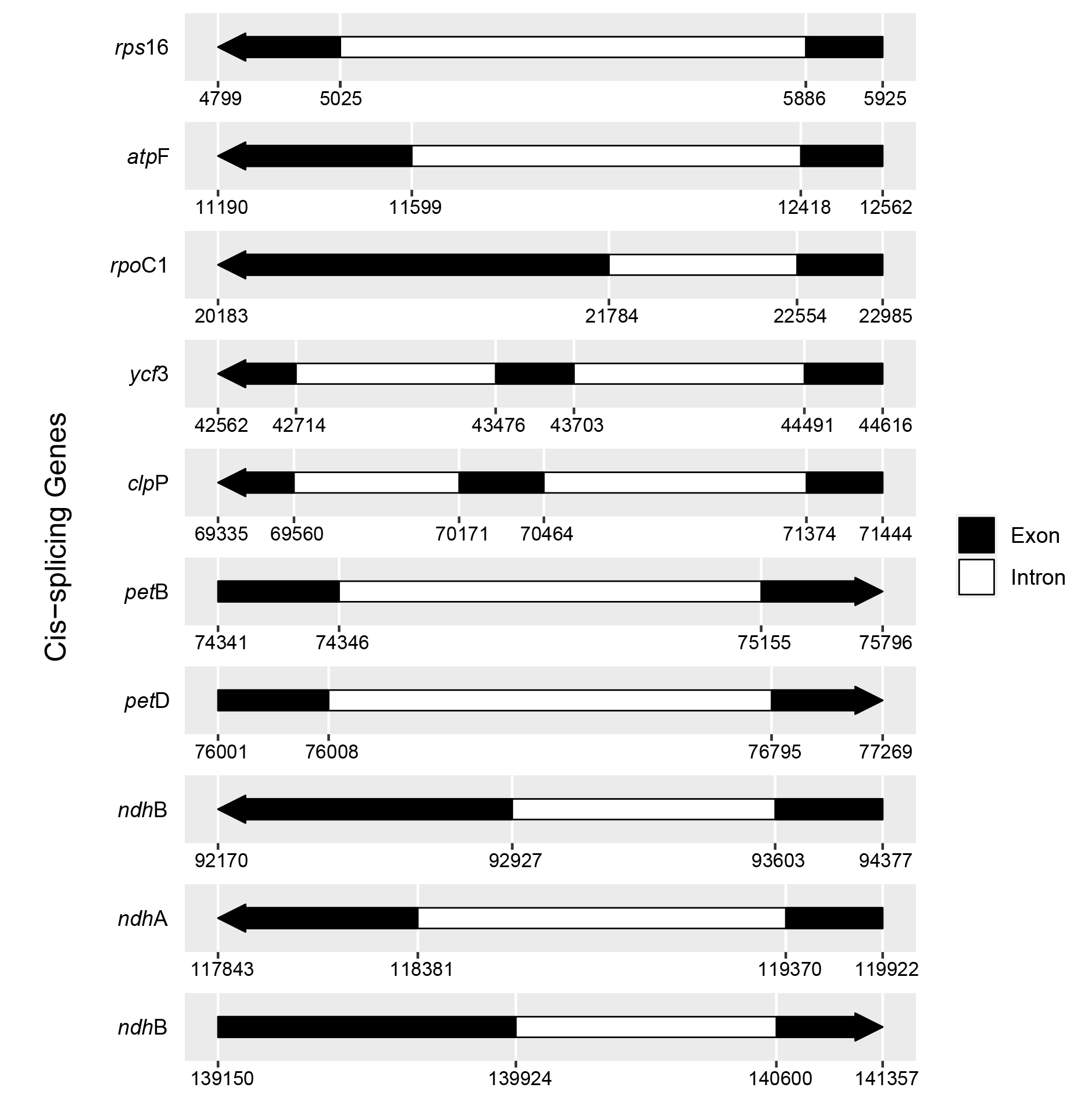


**A**


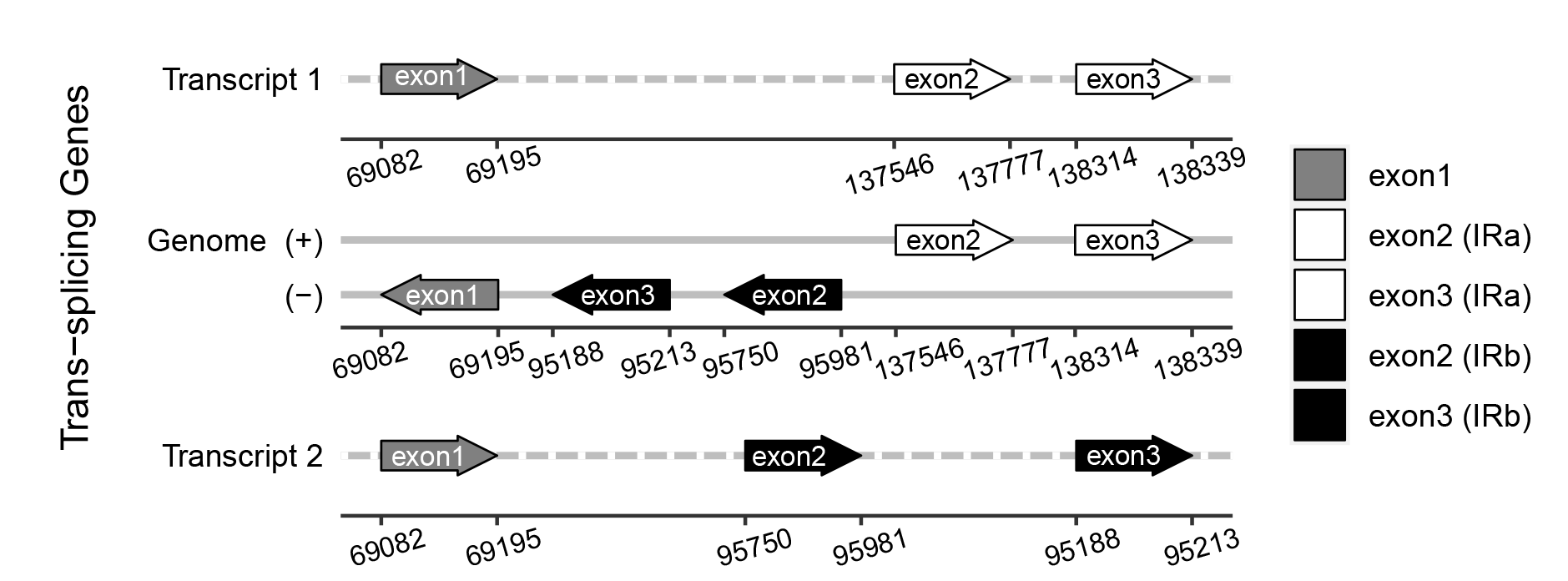


**B**

**Figure S2.** The 11 cis-splicing genes (A) and one trans-splicing gene *rps*12 (B) of the *Amaranthus roxburghianus* plastome.
